# Comparison of Injury Patterns between Electric Bicycle, Bicycle and Motorcycle Accidents

**DOI:** 10.3390/jcm10153359

**Published:** 2021-07-29

**Authors:** Emilian Spörri, Sascha Halvachizadeh, Jamison G. Gamble, Till Berk, Florin Allemann, Hans-Christoph Pape, Thomas Rauer

**Affiliations:** 1 Department of Trauma Surgery, University Hospital Zurich, 8091 Zurich, Switzerland; emilian.spoerri@gmail.com (E.S.); Sascha.Halvachizadeh@usz.ch (S.H.); till.berk@usz.ch (T.B.); Florin.Allemann@usz.ch (F.A.); hans-christoph.pape@usz.ch (H.-C.P.); 2 St. George’s University School of Medicine, St. George, Grenada; jg120gamble@gmail.com

**Keywords:** E-bike injuries, polytrauma, outcome, injury pattern comparison

## Abstract

Background: Electric bicycles (E-bikes) are an increasingly popular means of transport, and have been designed for a higher speed comparable to that of small motorcycles. Accident statistics show that E-bikes are increasingly involved in traffic accidents. To test the hypothesis of whether accidents involving E-bikes bear more resemblance to motorcycle accidents than conventional bicyclists, this study evaluates the injury pattern and severity of E-bike injuries in direct comparison to injuries involving motorcycle and bicycle accidents. Methods: In this retrospective cohort study, the data of 1796 patients who were treated at a Level I Trauma Center between 2009 and 2018 due to traffic accident, involving bicycles, E-bikes or motorcycles, were evaluated and compared with regard to injury patterns and injury severity. Accident victims treated as inpatients at least 16 years of age or older were included in this study. Pillion passengers and outpatients were excluded. Results: The following distribution was found in the individual groups: 67 E-bike, 1141 bicycle and 588 motorcycle accidents. The injury pattern of E-bikers resembled that of bicyclists much more than that of motorcyclists. The patients with E-bike accidents were almost 14 years older and had a higher incidence of moderate traumatic brain injuries than patients with bicycle accidents, in spite of the fact that E-bike riders were nearly twice as likely to wear a helmet as compared to bicycle riders. The rate of pelvic injuries in E-bike accidents was twice as high compared with bicycle accidents, whereas the rate of upper extremity injuries was higher following bicycle accidents. **Conclusion:** The overall E-bike injury pattern is similar to that of cyclists. The differences in the injury pattern to motorcycle accidents could be due to the higher speeds at the time of the accident, the different protection and vehicle architecture. What is striking, however, is the higher age and the increased craniocerebral trauma of the E-bikers involved in accidents compared to the cyclists. We speculate that older and untrained people who have a slower reaction time and less control over the E-bike could benefit from head protection or practical courses similar to motorcyclists.

## 1. Introduction

E-bikes, marketed as a clean alternative to cars with low energy consumption, have become a popular mode of transport with increasing interest [1]. Due to an increasing number of E-bike accidents [2], the topic of accident prevention and safety has been raised too. In Switzerland, the percentage of E-bikes sold as a percentage of all bicycles sold increased from 3.9% in 2008 to 32.2% in 2018 [3]. The trend started in China, where about 90% of all E-bikes were registered in 2011 [4] and where most of the early studies on E-bike accidents originated [5,6,7].

According to the motor assistance, E-bikes can reach speeds of up to 25 km/h or 45 km/h, hence they are able to reach higher velocities with less effort when compared with conventional bicycles [8]. Accordingly the rise in number and use of E-bikes led to a substantial increase in E-bike related traffic accidents [9]. Some studies have focused on crash characteristics [10], experience surveys [10,11] or riding behavior [12]. Others dealt with injury severity [1,13,14,15,16] or injury patterns occurring from E-bike accidents [9,13,14,15,16].

To date, only a few studies comparing E-bike related injuries with those suffered by conventional bicyclists or motorcyclists are available. A previous study, evaluating the injury severity of E-Bikers compared to conventional bicyclists, in police-recorded accidents without comparison of injury patterns, showed diverging results in injury severity [1]. 

The aim of this study was to evaluate the patterns and severity of E-bike injuries in comparison to findings in conventional bicycle and motorcycle accidents to further fill the gap of available literature on E-bike injuries. It is hypothesized that E-bike accidents have more similarity with motorcycle accidents than with conventional bicyclists due to higher speed.

## 2. Materials and Methods

This study was designed as a monocentric retrospective cohort study and was approved by the local institutional review board (PB_2016-01888). It follows the Strengthening the Reporting of Observational Studies in Epidemiology (STROBE) guidelines for reporting observational studies [17].

### 2.1. Setting

This study includes patients that were treated due to a road traffic accident at an academic Level I Trauma Center between 2009 and 2018. All medical data were collected from the electronical medical records during the hospitalization and analyzed retrospectively. Patients were followed-up until discharge from the hospital. 

### 2.2. Inclusion and Exclusion Criteria

Patients were included in this study if they were treated following a road traffic accident including E-bikes, bicycles or motorcycles. Further, patients were 16 years and older. Patients who were hit by a bike, or pillion passengers were excluded from this study. Patients’ data with more than 10% missing values were excluded from this study. Further, patients with injuries resulting from a motorized standing scooter accident were excluded. Patients who had an accident abroad were excluded, with the exception of patients transferred to our hospital from neighboring countries within 24 h of the accident. Patients who underwent elective surgery after a bike accident without first presenting to our hospital for initial treatment were also excluded. Outpatients were excluded due to a lack of detailed information on accident mechanism and medical clarification.

All patients were stratified according to the vehicle driven during the injury into: Group E-Bike (E), Group Bicycle (B), or Group Motorcycle (M).

The bicycle group contained conventional bicycles and mountain bikes. The E-Bike group included both E-Bikes with motor assistance up to 25 km/h and those with assistance up to 45 km/h, as it was not possible to retrospectively distinguish between these two types of assistance from the available data set. In the group of motorcycles, in addition to classic motorcycles, mopeds were also included. 

### 2.3. Search Strategy

The patients were identified using the appropriate International Classification of Diseases (ICD) Code of transportation accidents (ICD V99) in the computerized patient database. From this pool only conventional bicycle, E-bike and motorcycle accidents were selected. The medical database enabled instantaneous retrieval of past diagnostic reports, scores, treatment and other relevant documents to analyze. Knowing that not all possible rider accidents had the right ICD Code nor every report had accurate information about vehicle or accident type, patients with incomplete documentation were called and interviewed. 

### 2.4. Data Collection

Data collected included sex, age, helmet use, collision or self-accident, anatomic region of injury, injury severity regarding the Injury Severity Score (ISS) [18], dislocated/open fractures regarding the radiology report and initial Glasgow Coma Scale (GCS) [19]. Size data were asked for during hospital stay. Outcomes included treatment, intensive care unit (ICU), mortality and duration of hospital stay. Early onset surgery and late onset surgery, which was performed at least 48 h after the accident respectively planned as an elective procedure, were summarized in one group. Anatomic regions were divided to upper extremity, lower extremity, thorax, abdomen, pelvis, spine (cervical, thoracic, lumbar, sacral), head, face and skin. According to the ISS [18] the injuries were classified as minor or major trauma. The cut-off point for a major trauma was settled as ISS over 15. Traumatic brain injuries (TBI) were further classified into mild (initial GCS-Score 13–15), moderate (initial GCS-Score 9–12) and severe (initial GCS-Score 3–8).

### 2.5. Statistical Analysis

The primary analysis of this work bases on descriptive statistics in order to present comparative measures among the three groups. Continuous variables are presented as mean with standard deviation (SD), categorical variables as numbers and percentage. Comparison of the three groups (E/B/M) was initially performed with Kruskal Wallis test in cases, where data distribution appeared nonuniform. The additional risk of suffering from specific injuries were calculated and presented with odds ratio (OR) and 95% confidence interval (95% CI). 

A *p*-value of less than or equal to 0.05 was considered as statistically significant. Statistical analysis was performed using R (R Core Team (2020). R: A language and environment for statistical computing. R Foundation for Statistical Computing, Vienna, Austria. URL https://www.R-project.org/ accessed on 28 July 2021).

## 3. Results

Out of 3932 eligible patients, 1796 met the inclusion criteria, 67 (3.7%) had E-bike related injuries, 1141 (64%) had bicycle related injuries and 588 (33%) had motorcycle related injuries.

The average age at the time of injury was 56 years for E-bikers, which was the oldest group by far, compared to 42 years for the bicyclists and 41 years for the motorcyclists. The male-to-female ratio in total was 3.2 to 1. Motorcyclists had the highest male rate with 88% (*n* = 515) followed by the bicyclists with 72% (*n* = 816) and the E-bikers with the lowest of 61% (*n* = 41, Table 1).

Motorcyclists had the significantly highest collision proportion with 41% (*n* = 243). The lowest collision proportion were the E-bikers with only 13% (*n* = 9), meaning they had the highest self-accident proportion. Helmet use was established for 73% (*n* = 49) of the E-bikers whereas only 38% (*n* = 429) of the bicyclists wore a helmet. Generally, motorcyclists almost always wore a helmet 96% (*n* = 243), as it is mandatory in Switzerland. Standing out in terms of injury severity with the highest rate in major traumas 39% (*n* = 230), dislocated fractures 60% (*n* = 353), open fractures 16% (*n* = 96), paralysis 2.9% (*n* = 17) and mortality 2.6% (*n* = 15) were the motorcyclists (Table 2). E-bike driver were more commonly subject of collison, rather than self-inflicted accidents as compared with bicyclists (OR 1.64, 95%CI 1.31 to 2.1, *p* < 0.001).

Head injuries were seen more often in bicycle (64%) and E-bike (66%) accidents compared to motorcycle accidents with 47%. Motorcyclists also had the lowest rate of facial injuries at 19%, compared with bicyclists and E-bikers with 42% and 40% respectively. On the other hand, lower extremity (55%), thoracic (51%), abdominal (18%) and spinal injuries (24%) were all significantly more frequent in motorcyclists. Upper extremity injuries (48% and 41%) were more prevalent than lower extremity injuries (28% and 25%) in E-bikers and bicyclists. In contrast, motorcyclists had an antipodal rate with a higher frequency of lower extremity injuries (55%) than upper extremity injuries (43%). The injury localization shows clear similarities between E-bikers and conventional bicyclists. Exceptions can be recognized in terms of thoracic and pelvic injuries. Bicyclists have a higher rate in thoracic injuries (37% vs. 25%) whereas E-bikers were more likely to experience pelvic injuries (13% vs. 7%). The risk of suffering from a pelvic injury was nearly twice as high following an E-bike accident when compared with bicycle (OR 1.95, 95%CI 1.01 to 4.08, *p* = 0.0093). However, E-bike driver were less likely to suffer from upper extremity injuries when compared with bicyclists (OR 0.80, 95%CI 0.67 to 0.98, *p* = 0.035). A complete list of injury localization is provided in Table 3.

Motorcyclists had the fewest traumatic brain injuries (TBI) respectively (resp.) head injuries with 47% compared to E-bikers with 66% and bicyclists with 64%. The rate of moderate TBI was found to be significantly higher among E-bikers at 10%. In addition, the initial GCS-Score of less than 15 at the accident site was seen the most in the E-bike group by far. For more details about traumatic brain injury see Table 4.

Motorcyclists were found to have a hospital stay nearly twice as long (10 days) as compared to bicyclists (5 days) and E-bikers (6) days. The same pattern is seen for surgical procedures required (74%) and intensive care unit stay (ICU) required (29%) due to consequences of the initial accident only, where motorcyclists clearly take the lead. Wound care without surgery after an E-bike accident was performed in 48% of the cases, 43% after a bicycle accident and 33% after a motorcycle accident. Following a hospital stay 10% of the bicyclists, 12% of the E-bikers and 27% of the motorcyclists went to a stationary rehabilitation center. Only a few patients were transferred to another hospital respectively regionalized (Table 5).

## 4. Discussion

The main aim of this study was to investigate the characteristics of E-bike accidents compared to bicycles and motorcycles with the question of wether the injury pattern of E-Bike accidents is more similar to that of motorcycle accidents or more similar to that of bicycle accidents.

This study has revealed the following main results:

1. Injury patterns in E-bike accidents are more comparable to those of bicyclists than to those of motorcyclists.

2. The rate of pelvic injuries in E-bike accidents is twice as high compared with bicycle accidents, whereas the rate of upper extremity injuries was higher following bicycle accidents.

3. E-bikers who sustained injuries were older than bicycle or motorcycle riders.

Technology has produced a number of recreational vehicles over the past few decades. The literature follows exposing the dangers and pitfalls of riding without proper safety precautions in most vehicle types. Attention has now turned to a novel class of two-wheel vehicle. As the scientific community works to catch up with the fast-paced rate of technological advancements in transportation technologies such as E-bikes, trends in the data, such as those presented in this study, will lay the groundwork to educate, advise and eventually enact policies and precautions aimed at reducing and preventing E-bike related injuries. Another major topic is the growing availability of bike sharing programs, especially E-bike sharing. Gross et al. [14], Baschera et al. [16] and DiMaggio et al. [9] compared E-bike accidents with other two-wheel vehicular related traumas. Gross et al. [14] compared resulting injuries between children and adults from E-bike accidents whereas, Baschera et al. [16] compared traumatic brain injuries caused by E-bike and bicycle accidents. 

In accordance with the results of Gross et al. [14] the assumption was that E-bike accidents are similar to motorcycle accidents in terms of injury patterns. However, the results of this study could not reproduce these results and showed conversely that the injury patterns in E-bike accidents are more comparable to those of bicyclists than to those of motorcyclists. E-bike users, reported to the hospital, are less frequently male as compared with conventional bicycle and motorcycle users. We found that the majority of cases involved middle-aged victims, which is in accordance with previous studies [1,13,16]. Whereas conventional bicycle or motorcycle accident victims were found to be considerably younger [20]. Self-accidents of E-bikers were higher than those seen with bicyclists and motorcyclists, which may be attributed to higher age, likely longer reaction times and a higher mental workload in difficult traffic situation [8]. Due to the retrospective nature of the present study, no statements could be made regarding either the speed of the E-bike riders at the time of the accident or any accident partners in non-self-inflicted accidents due to a lack of documentation. This limits the generalizability and significance of the results of the present study. In line with a recent study confirming that e-bikers ride faster than conventional cyclists [8], this could also be assumed for the present study and could account for an influence on injury distribution. Greater TBI rates in E-bikers compared to conventional bicyclists was found, despite the fact that E-bikers wore helmets almost twice as often as bicyclists. In addition, the E-bikers initial GCS-Score indicated abnormal resp. under 15 almost twice as often as bicyclists.

Overall, the injury patterns in E-bike accidents are more comparable to those of bicyclists than to those of motorcyclists. However, while the rates of spinal cord injury, severe traumatic brain injury, and upper extremity injury were comparable in all three groups, the rate of pelvic injury in E-bike accidents was comparable to that of motorcycle riders.

In this study, the percentage in E-bikers who experienced major trauma (19%) and patients requiring surgery (46%) was higher than that described by Papoutsi et al. (13% resp. 26%) and lower than Gross et al. regarding the percentage of major trauma (35%) [13,14]. Weiss et al. postulated the risk of an accident increases with age, but not with bicycle type [21]. This study confirms that this is different for higher or more severe accident rates. The percentage of TBI (66% in E-bikers and 64% in bicyclists) is very similar to the study of Baschera et al. (69% resp. 59%) [16]. Other studies have described TBI in under 40% of cyclists [6,22]. These big differences can be explained due to different definitions of TBI or data collection methods. The most common injury occurring in motorcycle accidents are lower extremity injury, in 55% of the cases, same incidence as in Fletcher et al. [23].

One strength of this study is that it was conducted at a Level I Trauma Center, with wide variations in injury severity. Victims from rural and urban accidents are included. The source of information is from a detailed patient database. Other studies relied on in field EMS personnel reports, insurance claim reports or questionnaire-based survey data-sets only. While the number of E-bike accidents may not be as high as other modes of transportation, it is directly proportional to bicycle and motorcycle accident rates, warranting further examination. An analysis of the national road traffic accident statistics showed a total of 20,022 patients involved in accidents in 2020: Of these, 3565 were motorcyclists, 1690 E-bike riders and 3637 bicyclists [24]. Given the retrospective nature of this study, it is possible that the number of E-bikers was under reported, thus not providing a completely clear picture on the actual number of e-bike users. Attempting to summarizing all two-wheeled vehicles in use on the streets in only 3 groups is a very pragmatic approach. Furthermore, this study was limited to adults 16 years of age or older, as children are treated at a separate hospital. Furthermore, being that this study was conducted at a Level 1 trauma center, it is not only a strength it is also an important limitation. It is possible that we saw a higher level of more severe E-bike related trauma than might be seen at lower-level trauma centers. E-biker with less serious injuries may have been treated at hospitals with more limited trauma care, resulting in a selection bias for more serious injuries in this study. In this study, patients from rural and urban settings of a major European city were included. Types of injury may be different with different terrain and traffic in other parts of the country, which may also represent a selection bias.

Future studies should include data from hospitals of varying trauma center levels.

## 5. Conclusions

The overall E-bike injury pattern is similar to that of cyclists. The difference in the injury pattern of motorcycle accidents could be due to the higher speeds at the time of the accidents, the different protective clothing and architecture of the vehicle. What is striking, however, is the higher age and the increased craniocerebral trauma of the E-bikers involved in an accident compared to the cyclists. In our opinion older and untrained people may have slower reaction times and less control over the E-bike, which are now faster due to the motorized support of the E-bike. This population could benefit from head protection or practical courses similar to that of motorcyclists. The innovation of environmentally friendly transportation brings benefits and novel, indisputable injury risks. Further studies are needed to compare the different types of E-bikes with more detailed data. Data from E-bike share companies would also bring more transparency in terms of the relationship between accidents and commercial use.

## Figures and Tables

**Table 1:** Demographics.

	Bicycle *n* = 1141	E-Bike *n* = 67	Motorcycle *n* = 588
Age (years) (SD)	42.1 (16.0)	56.0 (15.2)	40.8 (15.3)
BMI (kg/m^2^) (SD)	23.8 (3.4)	25.0 (3.8)	25.7 (4.1)
Sex (male) (%)	816 (71.5%)	41 (61.2%)	515 (87.6%)

**Table 2:** Accident Type and Severity.

	Bicycle *n* = 1141	E-Bike *n* = 67	Motorcycle *n* = 588
Collision	264 (23.1%)	9 (13.4%)	243 (41.3%)
Helmet use	429 (37.6%)	49 (73.1%)	564 (95.9%)
Major trauma	207 (18.1%)	13 (19.4%)	230 (39.1%)
Dislocated fractures	425 (37.2%)	30 (44.8%)	353 (60.0%)
Open fractures	31 (2.7%)	1 (1.5%)	96 (16.3%)
Paralysis	13 (1.1%)	1 (1.5%)	17 (2.9%)
Mortality	14 (1.2%)	1 (1.5%)	15 (2.6%)

**Table 3:** Injury Localization.

	Bicycle *n* = 1141	E-Bike *n* = 67	Motorcycle *n* = 588
Head	731 (64.1%)	44 (65.7%)	275 (46.8%)
Face	479 (42.0%)	27 (40.3%)	112 (19.0%)
Thorax	427 (37.4%)	18 (26.9%)	300 (51.0%)
Abdomen	81 (7.1%)	3 (4.5%)	107 (18.2%)
Pelvis	84 (7.4%)	9 (13.4%)	99 (16.8%)
Spine total	167 (14.6%)	11 (16.4%)	142 (24.1%)
Upper extremities	472 (41.4%)	32 (47.8%)	251 (42.7%)
Lower extremities	279 (24.5%)	19 (28.4%)	326 (55.4%)

**Table 4:** Traumatic Brain Injury.

	Bicycle *n* = 1141	E-Bike *n* = 67	Motorcycle *n* = 588
TBI total	731 (64.1%)	44 (65.7%)	275 (46.8%)
Mild TBI	645 (56.5%)	35 (52.2%)	213 (36.2%)
Moderate TBI	45 (3.9%)	7 (10.4%)	28 (4.8%)
Severe TBI	41 (3.6%)	2 (3.0%)	34 (5.8%)
Initial GCS <15	239 (20.9%)	26 (38.8%)	129 (21.9%)

**Table 5:** Hospitalization.

	Bicycle *n* = 1141	E-Bike *n* = 67	Motorcycle *n* = 588
Required ICU stay	148 (13.0%)	11 (16.4%)	173 (29.4%)
Required Surgery	590 (51.7%)	35 (52.2%)	434 (73.8%)
Wound care	491 (43.0%)	32 (47.8%)	196 (33.3%)
Hospital stay (days) (SD)	5.0 (6.7)	5.9 (5.5)	10.2 (10.3)
Discharged home	993 (87.0%)	54 (80.6%)	385 (65.5%)
Rehabilitation	109 (9.6%)	8 (11.9%)	160 (27.2%)
Relocation/Transfer	25 (2.2%)	4 (6.0%)	28 (4.8%)

## Data Availability

Data is accessible on reasonable request.
